# Broader factors associated with current adolescent overweight/obesity status in YRBSS 2023: Survey-aware analysis and calibrated AI-assisted classification

**DOI:** 10.1371/journal.pone.0360155

**Published:** 2026-10-08

**Authors:** Thu Hoang Thanh, Nhan Nguyen Thi, Toan Nguyen Khac

**Affiliations:** 1 University of Economics, Ho Chi Minh City, Vietnam; 2 Department of Midwifery, Faculty of Nursing and Medical Technology, University of Medicine and Pharmacy, Ho Chi Minh City, Vietnam; 3 Institute of Intelligent and Interactive Technologies, University of Economics, Ho Chi Minh City, Vietnam; Shiraz University of Medical Sciences, IRAN, ISLAMIC REPUBLIC OF

## Abstract

Adolescent overweight and obesity are shaped by demographic, behavioral and psychosocial factors, but it remains unclear whether broader predictor information improves cross-sectional classification of current overweight/obesity status more consistently than changing the machine-learning algorithm. This cross-sectional secondary analysis used 2023 Youth Risk Behavior Surveillance System data from 17,814 United States adolescents aged 12–18 years. Survey-aware multivariable logistic regression was used to identify independent correlates, while Random Forest and XGBoost were evaluated under base and extended predictor frameworks. Models were assessed on a held-out test set for discrimination, threshold-dependent performance, calibration and interpretability. The prevalence of overweight/obesity was 31.8%. Male sex and Black or African American and Hispanic/Latino race/ethnicity were associated with higher odds of overweight/obesity, whereas higher moderate-to-vigorous physical activity and more frequent breakfast intake were associated with lower odds. Extended models outperformed their corresponding base models: ROC-AUC increased from 0.6241 to 0.6894 for Random Forest and from 0.6217 to 0.6724 for XGBoost. RF-Extended was selected for calibration and interpretation. Isotonic recalibration preserved discrimination while improving probability reliability, reducing the Brier score from 0.2126 to 0.1960 and expected calibration error from 0.1160 to 0.0213. Exploratory SHAP analysis identified sex, race/ethnicity, physical activity, sleep duration, age and breakfast frequency among the features contributing most strongly to RF-Extended output. Because predictors and BMI status were measured during the same survey cycle, the models classify current status and should not be interpreted as predicting future obesity. Any screening or prevention application, including potential use in Vietnamese adolescent populations, would require prospective and external validation.

## Introduction

Overweight and obesity among adolescents are major global public health challenges [1]. More than 390 million children and adolescents now live with overweight, including about 160 million with obesity [2,3]. This burden is likely to continue rising [4]. In the United States in particular, 19.7% of children and adolescents are currently living with obesity [5]. Adolescent overweight/obesity is also associated with many adverse consequences for cardiometabolic health, quality of life and mental health [6]. Public health therefore needs not only to describe the burden but also to characterize population groups that are more likely to have current overweight/obesity, which may inform future prevention research and resource planning.

Adolescent overweight/obesity is a multifactorial condition shaped by the interaction of biological, behavioral, environmental and social factors [6–8]. Alongside demographic factors, modifiable factors such as physical activity, breakfast, sleep and other behavioral/psychosocial variables may contribute to the observed cross-sectional pattern of overweight/obesity [9–11]. Machine-learning methods have increasingly been investigated for obesity classification and risk modeling, with recent systematic reviews evaluating their predictive performance and, in some cases, comparing them with conventional regression approaches [12,13]. More recently, studies focused specifically on adolescents have incorporated machine-learning and interpretable modeling approaches. For example, a 2021 YRBSS analysis combined Random Forest and XGBoost with SHAP to characterize behavioral features contributing to obesity classification [14], while other recent studies have applied interpretable machine-learning frameworks in adolescent populations [15,16]. Nevertheless, important methodological gaps remain regarding calibration, survey-aware epidemiologic inference, transparent internal validation, and the distinction between model-based feature contributions and independent epidemiologic correlates. For public health, the key gap is not only which model performs better [17] but whether enriching the predictor framework within the range of available variables can improve classification of current overweight/obesity status more consistently than changing the algorithm alone. In this context, calibration and interpretability are especially important because model-estimated probabilities of current overweight/obesity should be reliable and interpretable if they are to inform future supportive applications [18,19].. Model evaluation also needs to be placed within an equity-aware framework because performance and the practical meaning of model outputs may not be uniform across population subgroups.

Using Youth Risk Behavior Surveillance System (YRBSS) 2023 [20], this study combines survey-aware multivariable logistic regression [21] and machine-learning classification to address three questions: (1) which independent correlates are associated with current adolescent overweight/obesity; (2) whether expanding the predictor framework with behavioral and psychosocial variables available in YRBSS improves calibrated cross-sectional classification more consistently than changing model class alone; and (3) whether the selected model can generate sufficiently calibrated and interpretable probability estimates of current overweight/obesity status. Because the predictors and BMI status were measured during the same survey cycle, the machine-learning component is explicitly cross-sectional and does not predict future onset of overweight/obesity. Potential screening or prevention applications are considered only as future possibilities requiring prospective and external validation.

## Methods

### Study data and participants

This study was a cross-sectional secondary analysis using publicly available data from the 2023 YRBSS. The data were accessed for research purposes on 10 November 2025. The original dataset included 20,103 respondents. Because BMI-for-age percentile (BMIPCT) was required to define the study outcome, 2,289 respondents with missing BMIPCT were excluded (11.4% of the original sample), leaving a final analytic sample of 17,814 adolescents aged 12–18 years. Basic demographic characteristics of respondents included in and excluded from the analytic sample were compared to assess the potential for selection bias, with results reported in S11 Table. The participant flow is summarized in Fig 1. YRBSS data are publicly available and de-identified.

**Fig 1:**
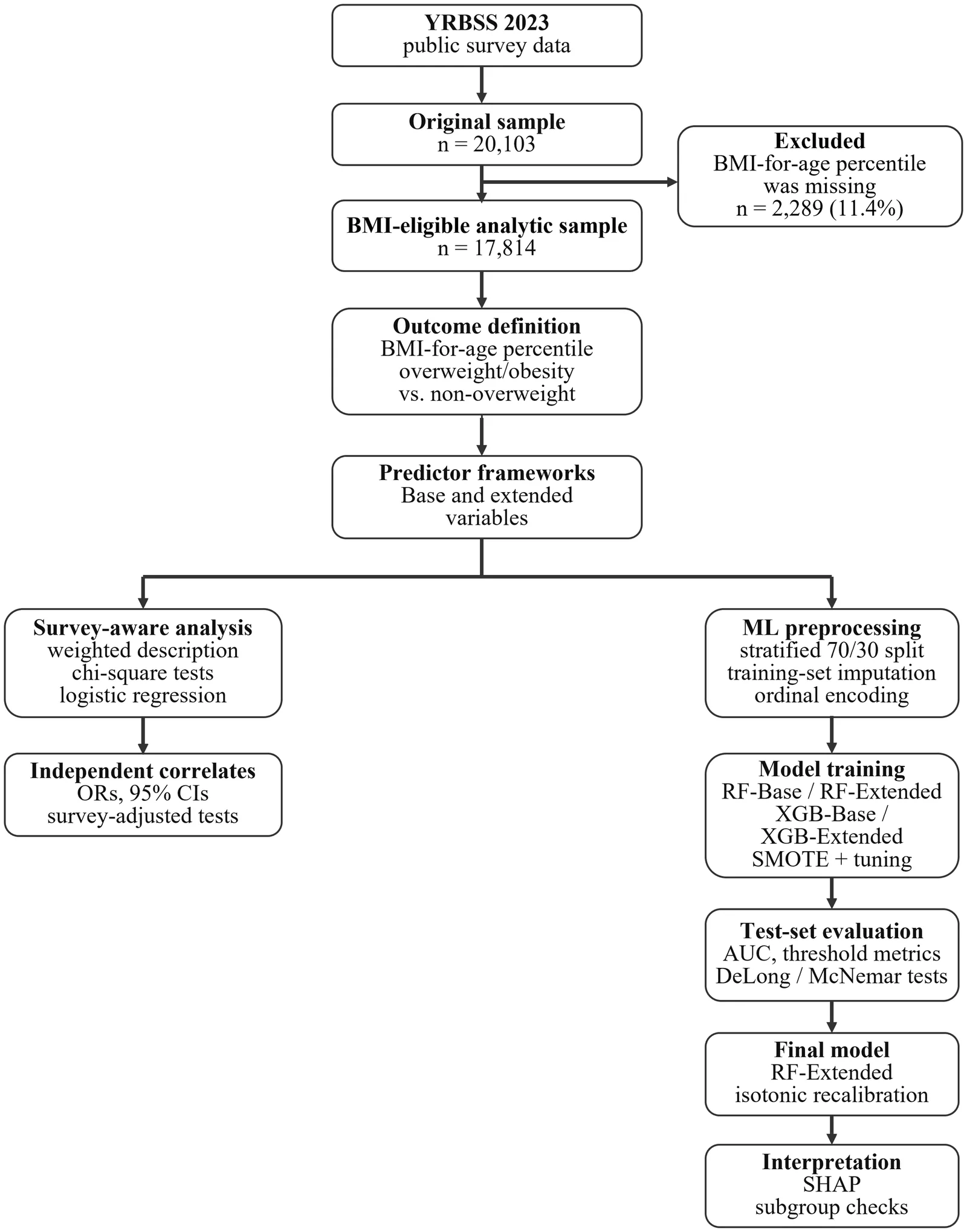
Participant flow and missing-data analysis framework.

### Ethics statement

Ethical approval for this secondary analysis was obtained from the Ethics Committee in Biomedical Research of the University of Medicine and Pharmacy at Ho Chi Minh City, approval No. 4232/DHYD-HDDD. For the original 2023 YRBS data collection, the survey protocol was approved by the institutional review boards of CDC and ICF. Student participation was anonymous and voluntary, and local parental permission procedures were followed before survey administration [22]. The present study used publicly available, de-identified YRBSS data and involved no direct contact with participants. Therefore, the authors did not recruit participants or obtain additional informed consent for this secondary analysis.

### Survey design, outcome and predictor frameworks

YRBSS uses a complex multistage survey design [23]. In this study, descriptive analyses and logistic regression were implemented in a survey-aware manner, accounting for survey weights and survey design. The machine-learning component was conducted as a complementary analysis on the analytic sample to evaluate cross-sectional classification of current overweight/obesity status rather than weighted population inference. Weight status was defined using the CDC sex-specific BMIPCT. In descriptive analyses, the outcome was presented in three categories including non-overweight, overweight and obese. Non-overweight combined underweight and normal weight. In inferential analyses and machine-learning classification, the main outcome was defined as a binary overweight/obesity variable, in which the positive group included overweight or obese participants and the reference group was non-overweight. This grouping was used for cross-sectional classification and comparability across models rather than clinical differentiation between overweight and obesity as two separate endpoints.

The analytic framework included two nested predictor configurations including a base framework and an extended framework. Predictors included age, sex, race and behavioral and psychosocial factors related to adolescent overweight/obesity. The base framework included core predictors commonly used in YRBSS analyses of overweight/obesity. The extended framework retained all predictors from the base framework and added broader behavioral and psychosocial variables available in YRBSS 2023. Variable definitions and category coding rules were prespecified before model development, and detailed variable definitions and coding are provided in S1 Table. The overall study design and analytical workflow are summarized in Fig 1. This schematic illustrates the progression from the YRBSS 2023 source population to outcome definition, survey-aware epidemiologic analysis, machine-learning model development, calibration, and interpretability assessment.

For machine learning, predictors were encoded according to their measurement scale. Ordinal variables with a meaningful natural order, including age, physically active days, sleep duration, breakfast frequency, dietary-frequency variables, substance-use frequency and mental-health frequency, were represented using ordered integer codes that preserved the natural category sequence. Nominal variables without an inherent ordering, including race/ethnicity and tobacco-cessation status, were one-hot encoded so that no artificial numerical ordering was imposed. Sex was represented using a binary indicator (Female = 0, Male = 1). The variable-specific coding and encoding scheme is provided in S1 Table.

### Data preparation and association analysis

For machine learning, missing predictors were handled by simple imputation in the preprocessing pipeline. Missing age was imputed using the median category of the training set, missing sex was imputed using the training-set mode and the remaining predictors were imputed using group-mode imputation by age-by-sex strata estimated from the training set. If a stratum-specific mode could not be determined, the overall training-set mode was used. All imputation parameters were derived from the training set and then applied unchanged to the testing set to avoid information leakage.

The extent of missingness was quantified for each predictor before imputation and is reported in S12 Table. To assess whether the machine-learning findings were materially influenced by the imputation procedure, a complete-case sensitivity analysis was additionally performed for RF-Extended. This analysis retained only participants with observed values for all 16 predictors in the final RF-Extended model, resulting in 8,739 complete cases (49.1% of the BMI-eligible analytic sample). The same RF-Extended model specification and evaluation framework were retained, and performance was compared with the primary imputed analysis (S13 Table).

Sample characteristics were described across the three outcome groups and compared using survey-adjusted chi-square tests [24]. Independent correlates of the binary overweight/obesity outcome were then assessed using two survey-aware multivariable logistic regression (LR) models, a base model and an extended model. Results are reported as odds ratios (ORs), 95% confidence intervals (CIs) and two-sided p-values, with *p* < 0.05 considered statistically significant. The role of this part was to identify independent correlates and support public-health-relevant interpretation.

### Model development, evaluation and interpretation

The study implemented two tree-based methods, Random Forest (RF) [25] and XGBoost (XGB) [26], under four configurations, including RF-Base, RF-Extended, XGB-Base, and XGB-Extended. The aim was to assess whether expanding the predictor framework improved cross-sectional classification performance more consistently than switching algorithms alone. The dataset was split into a training set (70%) and a testing set (30%) using stratified sampling with a fixed random seed of 422. The train–test split was performed before any data-dependent preprocessing. The predefined ordinal, binary and one-hot encoding rules were applied consistently to the training and test sets, while imputation parameters were estimated from the training data only and applied unchanged to the held-out test set; no information from the test set was used during preprocessing or model development. Hyperparameter tuning was performed exclusively within the training data using Optuna with five-fold stratified cross-validation. For each model configuration, 500 trials were conducted, and the mean weighted F1 score across the five validation folds was used as the optimization objective. The hyperparameter search spaces and final selected values are provided in S8 Table. During cross-validation, SMOTENC was applied independently within each training fold and was not applied to the corresponding validation fold or the held-out testing set. SMOTENC was applied before model-specific encoding so that categorical predictors remained categorical during synthetic sampling; the ordinal, one-hot and binary encodings described above were then applied for model fitting. No additional class weighting was applied to either RF or XGB. To assess whether the main findings were materially dependent on the imbalance-handling strategy, a sensitivity analysis was conducted for the two extended models, RF-Extended and XGB-Extended. Each model was refitted without oversampling and without class weighting, while retaining the same predictor set, preprocessing sequence, model specification, and held-out test evaluation procedure as in the primary analysis. Performance was compared with the corresponding SMOTENC-based models using ROC-AUC, accuracy, sensitivity, specificity, and weighted F1 score (S14 Table). After hyperparameter selection, model stability was assessed within the training data using five-times repeated five-fold stratified cross-validation, yielding 25 validation folds. The final hyperparameter specification for each model was held fixed during this analysis. Performance stability was summarized using the mean and standard deviation of ROC-AUC, weighted F1, and positive-class F1 across resamples, with results reported in S9 Table. To further assess model convergence and potential overfitting, training-size learning curves were generated for all four model configurations using weighted F1 score, consistent with the optimization objective used during hyperparameter tuning. Learning curves were evaluated at increasing proportions of the available cross-validation training data (20%, 40%, 60%, 80%, and 100%). At each training size, weighted F1 was calculated separately for the training observations and the corresponding cross-validation validation observations. The final hyperparameter specification for each model was held fixed during learning-curve evaluation. The resulting training and cross-validation curves are presented in S15 Fig. To provide a direct assessment of model fit and potential overfitting, parallel performance summaries were generated for the original training observations and the held-out test set for all four model configurations. Absolute confusion-matrix counts, including true positives (TP), false negatives (FN), true negatives (TN), and false positives (FP), were reported together with ROC-AUC and weighted F1 score. Training-set confusion matrices were calculated on the original training observations rather than on synthetic observations generated by SMOTENC. Training-set results were used only as diagnostic measures of model fit; the primary performance estimates remained those obtained from the held-out test set (S16 Table). The preprocessing and model-development sequence is summarized in S17 Fig. Performance on the held-out testing set was evaluated using ROC-AUC and threshold-dependent operating characteristics, including accuracy, sensitivity, specificity, PPV, NPV and weighted F1-score. For the primary model comparison, accuracy-oriented operating thresholds were determined using validation data within the training partition and fixed before evaluation on the held-out testing set. For the selected RF-Extended model, the corresponding threshold on the raw probability scale was 0.564; Table 3 reports the primary test-set performance at this pre-calibration operating point.

After model selection, RF-Extended was recalibrated using isotonic regression based on validation data within the training partition. Isotonic recalibration changed the probability scale but largely preserved probability ranking. The accuracy-oriented RF-Extended threshold corresponded to 0.439 on the calibrated probability scale. A secondary sensitivity-oriented operating point was also prespecified to support a screening-oriented analysis. For RF-Extended, the sensitivity-oriented threshold was 0.428 before calibration and 0.304 after isotonic recalibration, with the calibrated threshold selected to target sensitivity as close as possible to the prespecified value of 0.70. The model specification, isotonic calibrator and operating thresholds were fixed before application to the held-out testing set. Calibration results are reported in S7 Table, while the threshold trade-off for RF-Extended is reported in S10 Table. Differences in AUC between model pairs were tested using the DeLong test [27], while differences in binary classification labels at the primary operating thresholds were assessed using McNemar’s test [28].

To interpret model behavior, feature importance was assessed using Mean Decrease Gini for RF and Gain for XGB. SHapley Additive exPlanations (SHAP) [29] were used as an exploratory model-interpretation analysis to characterize how individual features contributed to RF-Extended model output. The final RF-Extended model was fitted using the development data only, and SHAP values were subsequently calculated for observations in the held-out test set after completion of the primary model evaluation. SHAP values were interpreted solely as explanations of fitted-model behavior and not as independent associations, causal effects, or determinants of overweight/obesity. Survey-aware LR was conducted in SPSS version 27.0. Data processing, RF/XGB model development, model evaluation, calibration, visualization and SHAP analyses were implemented in Python 3.11.23 using conda 25.5.1. Key packages included numpy 2.0.2, pandas 2.3.1, scipy 1.13.1, scikit-learn 1.6.1, imbalanced-learn 0.12.4, statsmodels 0.14.0, xgboost 2.1.4, optuna 4.6.0, matplotlib 3.9.4, seaborn 0.13.2, shap 0.49.1 and openpyxl 3.1.5. The analysis code is publicly available at https://github.com/Khactoan240/Obesity; the exact release and commit corresponding to the submitted analysis are specified in the Data Availability Statement.

## Results

### Sample characteristics and prevalence of overweight/obesity

Of the 20,103 respondents in the original YRBSS 2023 dataset, 2,289 (11.4%) were excluded because BMI-for-age percentile was unavailable, leaving 17,814 respondents in the primary analytic sample. The demographic composition of excluded respondents differed from that of included respondents for age, sex and race/ethnicity (all *p* < 0.001; S11 Table). For example, females accounted for 53.9% of excluded respondents compared with 48.6% of included respondents, whereas White adolescents accounted for 38.0% and 49.6%, respectively. Hispanic/Latino adolescents represented 33.2% of excluded respondents and 28.3% of included respondents. Before imputation, predictor missingness varied across variables (S12 Table). Among the 16 predictors included in RF-Extended, missingness ranged from 0% for age and sex to 31.59% for water intake. Other variables with relatively high missingness included breakfast frequency (28.25%), tobacco-cessation status (23.40%), soda intake (22.90%), potato intake (22.21%), green-salad intake (22.13%) and mental-health frequency (20.26%).

Table 1 presents selected key characteristics across weight categories. In the analytic sample, 31.8% of adolescents were classified as overweight or obese. At the descriptive level, statistically significant differences across weight groups were observed for sex, race, moderate-to-vigorous physical activity (MVPA) days and water intake frequency, whereas age group did not differ significantly across the three groups. Descriptively, overweight/obesity was more common in males. Across weight-status groups, the proportion of White adolescents decreased from 51.7% in the non-overweight group to 41.3% in the obese group, whereas the proportion of Hispanic/Latino adolescents increased from 30.4% to 32.7%. For physical activity, the proportion with no MVPA days increased from 13.7% to 18.8%, whereas the proportion achieving MVPA on all 7 days per week decreased from 27.3% to 18.4%. Full descriptive results are provided in S2 Table.

**Table 1:** Weighted characteristics of participants across weight-status groups.

Variable	Non-overweight (n = 12145)	Overweight (n = 2710)	Obese (n = 2959)	$\chi^{2}$	P value
Sex				76.839	< 0.001
Female	6133 (48.0%)	1360 (48.2%)	1158 (39.1%)		
Male	6012 (52.0%)	1350 (51.8%)	1801 (60.9%)		
Age				38.824	0.091
14 years old or younger	1502 (11.2%)	360 (12.8%)	379 (14.4%)		
15 years old	3390 (25.6%)	739 (25.7%)	760 (23.2%)		
16 years old	3217 (26.2%)	712 (27.4%)	730 (24.7%)		
17 years old	2678 (24.8%)	628 (22.6%)	737 (25.9%)		
18 years old or older	1358 (12.2%)	271 (11.5%)	353 (11.7%)		
Race				189.996	< 0.001
White	6462 (51.7%)	1274 (45.0%)	1254 (41.3%)		
Black or African American	1021 (12.1%)	272 (16.1%)	332 (17.2%)		
Hispanic/Latino	3254 (30.4%)	828 (29.3%)	966 (32.7%)		
All Other Races	1408 (5.8%)	336 (9.6%)	407 (8.7%)		
MVPA days				168.812	< 0.001
0 days	1567 (13.7%)	380 (16.0%)	502 (18.8%)		
1 day	751 (6.5%)	177 (7.0%)	211 (7.1%)		
2 days	972 (9.4%)	239 (8.6%)	305 (11.4%)		
3 days	1198 (10.7%)	300 (11.4%)	357 (11.6%)		
4 days	1113 (9.5%)	274 (10.2%)	310 (12.0%)		
5 days	1689 (14.1%)	362 (13.5%)	415 (14.9%)		
6 days	1055 (8.8%)	231 (9.3%)	160 (5.8%)		
7 days	3800 (27.3%)	747 (23.9%)	699 (18.4%)		
Water intake				74.613	0.004
Did not consume	251 (2.7%)	41 (2.2%)	68 (3.0%)		
1 to 3 times	710 (7.3%)	164 (7.4%)	203 (9.0%)		
4 to 6 times	1010 (11.2%)	212 (11.2%)	264 (12.6%)		
1 time per day	737 (8.4%)	135 (7.0%)	133 (5.6%)		
2 times per day	1334 (17.8%)	293 (16.0%)	288 (14.0%)		
3 times per day	1297 (17.1%)	314 (15.9%)	323 (13.8%)		
4 times per day	2819 (35.6%)	725 (40.2%)	865 (42.0%)		

MVPA = moderate-to-vigorous physical activity. Values are presented as unweighted counts and weighted percentages. P-values were obtained using survey-adjusted chi-square tests. Selected variables are shown in the main text, full comparisons are provided in S2 Table.

### Independent correlates from survey-aware regression models

Table 2 presents selected results from the survey-aware logistic regression analysis of overweight/obesity; full estimates are provided in S3 Table and S4 Table. In the base model, the most notable correlates were mainly demographic variables and core modifiable behaviors. Male sex was associated with higher odds of the outcome (OR = 1.33), while some race groups also showed clear increases, most notably Hispanic/Latino adolescents relative to the reference group (OR = 1.81). In contrast, the most favorable MVPA level was associated with lower odds of overweight/obesity (OR = 0.70) and breakfast frequency showed a broadly consistent protective pattern, with the lowest odds at 7 days (OR = 0.59).

**Table 2:** Survey-aware multivariable logistic regression of overweight/obesity for the base and extended frameworks.

Predictor	Category comparison	Base OR (95% CI)	Base p-value	Extended OR (95% CI)	Extended p-value
Base demographic correlates					
Age	ref: ≤14 years				
	15 years	0.76 (0.60–0.96)	0.022	0.74 (0.57–0.96)	0.024
	17 years	0.78 (0.61–0.99)	0.044	0.76 (0.59–0.98)	0.033
Sex	Ref: Female				
	Male	1.33 (1.16–1.52)	<0.001	1.34 (1.17–1.53)	<0.001
Race	ref: White				
	Black or African American	1.51 (1.20–1.88)	0.001	1.51 (1.18–1.93)	0.001
	Hispanic/Latino	1.81 (1.35–2.41)	<0.001	1.95 (1.44–2.65)	<0.001
	All Other Races	1.12 (0.93–1.34)	0.226	1.07 (0.91–1.27)	0.396
Core behavioural correlates					
MVPA days	Ref: 0 days				
	7 days	0.70 (0.55–0.90)	0.005	0.67 (0.52–0.87)	0.003
Sleep	ref: 4 hours or less				
	6 hours	1.16 (0.95–1.43)	0.144	1.32 (1.06–1.63)	0.013
Breakfast days	ref: 0 days				
	4 days	0.58 (0.47–0.71)	<0.001	0.60 (0.49–0.73)	<0.001
	5 days	0.79 (0.65–0.96)	0.017	0.79 (0.65–0.97)	0.023
	6 days	0.65 (0.48–0.88)	0.007	0.68 (0.49–0.93)	0.016
	7 days	0.59 (0.48–0.72)	<0.001	0.59 (0.47–0.75)	<0.001
Potatoes	ref: Did not consume				
	2 times per day	1.90 (1.12–3.20)	0.017	2.13 (1.23–3.72)	0.008
Additional correlates in the extended framework					
Mental health	ref: Never				
	Rarely	—	—	0.84 (0.67–1.06)	0.146
	Sometimes	—	—	0.87 (0.71–1.06)	0.16
	Most of the time	—	—	1.05 (0.84–1.32)	0.672
	Always	—	—	1.21 (0.91–1.60)	0.178
All tobacco product cessation	ref: Did not use tobacco products				
	Yes	—	—	1.40 (1.03–1.92)	0.035
	No	—	—	1.03 (0.77–1.38)	0.838
Water intake	ref: Did not consume				
	4 or more times per day	—	—	1.35 (0.93–1.95)	0.115

Reference categories are shown as “Ref”. ORs and 95% CIs were estimated using survey-aware multivariable logistic regression accounting for the YRBSS complex sampling design. Only selected estimates are shown in the main text, full results are provided in S3 Table and S4 Table.

When the predictor set was expanded, the main association structure remained broadly stable and did not alter the basic epidemiologic logic of the findings. The OR for male sex was almost unchanged between the base and extended models (1.33 vs 1.34), while the most favorable MVPA level retained its protective association (0.70 in the base model and 0.67 in the extended model) and the effect of breakfast was generally maintained. One notable change was that sleep became clearer in the extended model (OR = 1.32), whereas the additional variables provided only a small number of extra independent signals. Thus, the main value of the extended framework should be understood as broadening the set of correlates considered in a public-health-relevant way without disrupting the core correlates, rather than being judged only by the number of new coefficients that reached statistical significance.

### Added value of broader determinants for cross-sectional classification

Across both tree-based algorithms, the extended models outperformed their corresponding base models. In Random Forest, AUC increased from 0.6241 to 0.6894 and in XGBoost, from 0.6217 to 0.6724. These within-algorithm gains were statistically significant in pairwise comparisons, with detailed DeLong and McNemar results provided in S5 Table. RF-Extended was therefore selected as the final model for calibration and interpretation. Subgroup analysis showed moderate variation across sex and race groups, supporting an equity-aware interpretation of model outputs (S6 Table).

A sensitivity analysis was performed to assess the influence of the imbalance-handling strategy on the two extended models (S14 Table). For RF-Extended, omitting SMOTENC reduced ROC-AUC from 0.6894 to 0.6830, accuracy from 0.6486 to 0.6384, sensitivity from 0.5640 to 0.5251, and weighted F1 from 0.6571 to 0.6458, while specificity increased slightly from 0.6878 to 0.6907. For XGB-Extended, ROC-AUC decreased from 0.6724 to 0.6671, accuracy from 0.6492 to 0.6436, sensitivity from 0.5423 to 0.5050, and weighted F1 from 0.6565 to 0.6492, while specificity increased from 0.6987 to 0.7077. RF-Extended retained higher discrimination than XGB-Extended under both imbalance-handling strategies. Overall, SMOTENC modestly improved discrimination and positive-class sensitivity, whereas the relative ranking of the two extended models was unchanged. Furthermore, learning-curve analysis showed the expected convergence pattern across all four model configurations (S15 Fig). Training weighted F1 was higher than cross-validation weighted F1 at smaller training sizes, while the gap generally narrowed as the amount of training data increased. Cross-validation performance improved with increasing training size and approached a plateau for all models. At the largest training size, cross-validation weighted F1 was 0.621 for RF-Base, 0.656 for RF-Extended, 0.630 for XGB-Base, and 0.654 for XGB-Extended. The two extended models consistently achieved higher validation performance than their corresponding base models. Overall, the narrowing training–validation gap and plateauing validation curves did not indicate severe overfitting.

Parallel training- and test-set results are reported in S16 Table. Training weighted F1 scores were 0.6280 for RF-Base, 0.6810 for RF-Extended, 0.6400 for XGB-Base, and 0.6840 for XGB-Extended, compared with held-out test values of 0.6215, 0.6571, 0.6316, and 0.6565, respectively. Training ROC-AUC was also moderately higher than held-out test ROC-AUC across the four configurations. The train–test differences were relatively small, with somewhat larger gaps for the extended models, and were consistent with the convergence patterns observed in the learning-curve analysis. These results did not indicate severe overfitting. Absolute TP, FN, TN, and FP counts for both datasets are provided in S16 Table.

### Calibration of the selected final model

Calibration analysis showed that isotonic recalibration brought the RF-Extended curve closer to the ideal line as shown in Fig 2. AUC changed little after recalibration (0.6894 vs 0.6891), whereas probability reliability improved, with the Brier score decreasing from 0.2126 to 0.1960 and ECE from 0.1160 to 0.0213. Additional calibration metrics are provided in S7 Table. The accuracy-oriented RF-Extended threshold changed from 0.564 on the raw probability scale to 0.439 on the calibrated probability scale.

**Fig 2:**
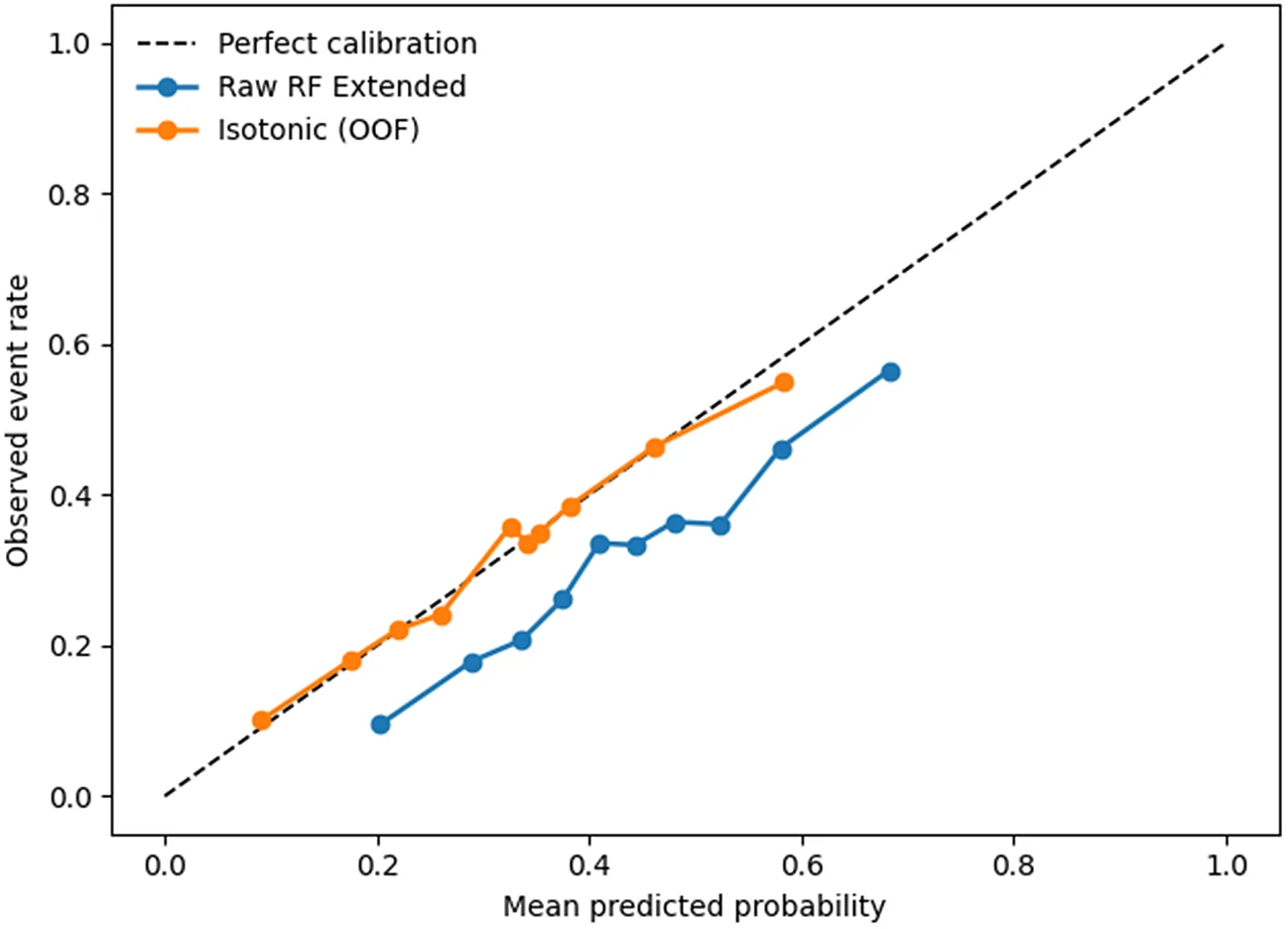
Calibration curves of the selected model (RF-Extended) before and after isotonic recalibration.

Table 3 reports the primary pre-calibration accuracy-oriented operating point, at which RF-Extended achieved an accuracy of 0.6486, sensitivity of 0.5640 and specificity of 0.6878. For the secondary sensitivity-oriented analysis, the RF-Extended threshold changed from 0.428 before calibration to 0.304 after isotonic recalibration. When the calibrated sensitivity-oriented threshold of 0.304 was applied unchanged to the held-out test set, RF-Extended achieved a sensitivity of 0.6841, with an accuracy of 0.6139, specificity of 0.5814, PPV of 0.4305, NPV of 0.7991 and weighted F1 of 0.6273 (S10 Table). This illustrates the expected trade-off between greater sensitivity and lower overall accuracy and specificity at a sensitivity-oriented operating point. Besides, the complete-case sensitivity analysis included 8,739 participants, corresponding to 49.1% of the BMI-eligible analytic sample. RF-Extended showed broadly similar performance in the complete-case and primary imputed analyses. ROC-AUC was 0.6788 in the complete-case analysis compared with 0.6894 in the primary analysis; corresponding values were 0.6396 versus 0.6486 for accuracy, 0.5601 versus 0.5640 for sensitivity, 0.6785 versus 0.6878 for specificity and 0.6472 versus 0.6571 for weighted F1 (S13 Table). These results indicate that the main model-performance pattern was not materially altered when the analysis was restricted to complete cases.

**Table 3:** Primary predictive performance of the base and extended RF and XGBoost models at accuracy-oriented operating points before isotonic recalibration.

Metric	RF-Base	RF-Extended	XGB-Base	XGB-Extended
AUC	0.6241 [0.6102, 0.6380]	0.6894 [0.6762, 0.7026]	0.6217 [0.6071, 0.6363]	0.6724 [0.6584, 0.6863]
Accuracy	0.6111	0.6486	0.6218	0.6492
Sensitivity	0.5252	0.5640	0.5367	0.5423
Specificity	0.6509	0.6878	0.6611	0.6987
PPV	0.4103	0.4553	0.4229	0.4543
NPV	0.7477	0.7732	0.7552	0.7674
Weighted F1	0.6215	0.6571	0.6316	0.6565

AUC = area under the receiver operating characteristic curve; PPV = positive predictive value; NPV = negative predictive value. Weighted F1 represents the support-weighted average of the class-specific F1 scores. Threshold-dependent metrics were calculated at accuracy-oriented operating points selected using validation data within the training partition and fixed before evaluation on the held-out test set. For RF-Extended, the raw probability threshold was 0.564.

### Exploratory interpretation of features contributing to model output

Fig 3 presents the exploratory SHAP summary plot for RF-Extended based on observations in the held-out test set. Sex and race/ethnicity showed the largest overall contributions to model output, followed by MVPA days, sleep duration, age, breakfast frequency, mental health and water intake. Fruit, green-salad, soda and potato intake, tobacco-cessation status, electronic-vapor use and marijuana use showed smaller overall contributions. The horizontal distribution of SHAP values indicates how individual feature values shifted the fitted model output toward or away from the overweight/obesity class. These patterns describe the behavior of RF-Extended and should not be interpreted as independent associations, causal effects, or evidence that changing a given feature would alter overweight/obesity status.

**Fig 3:**
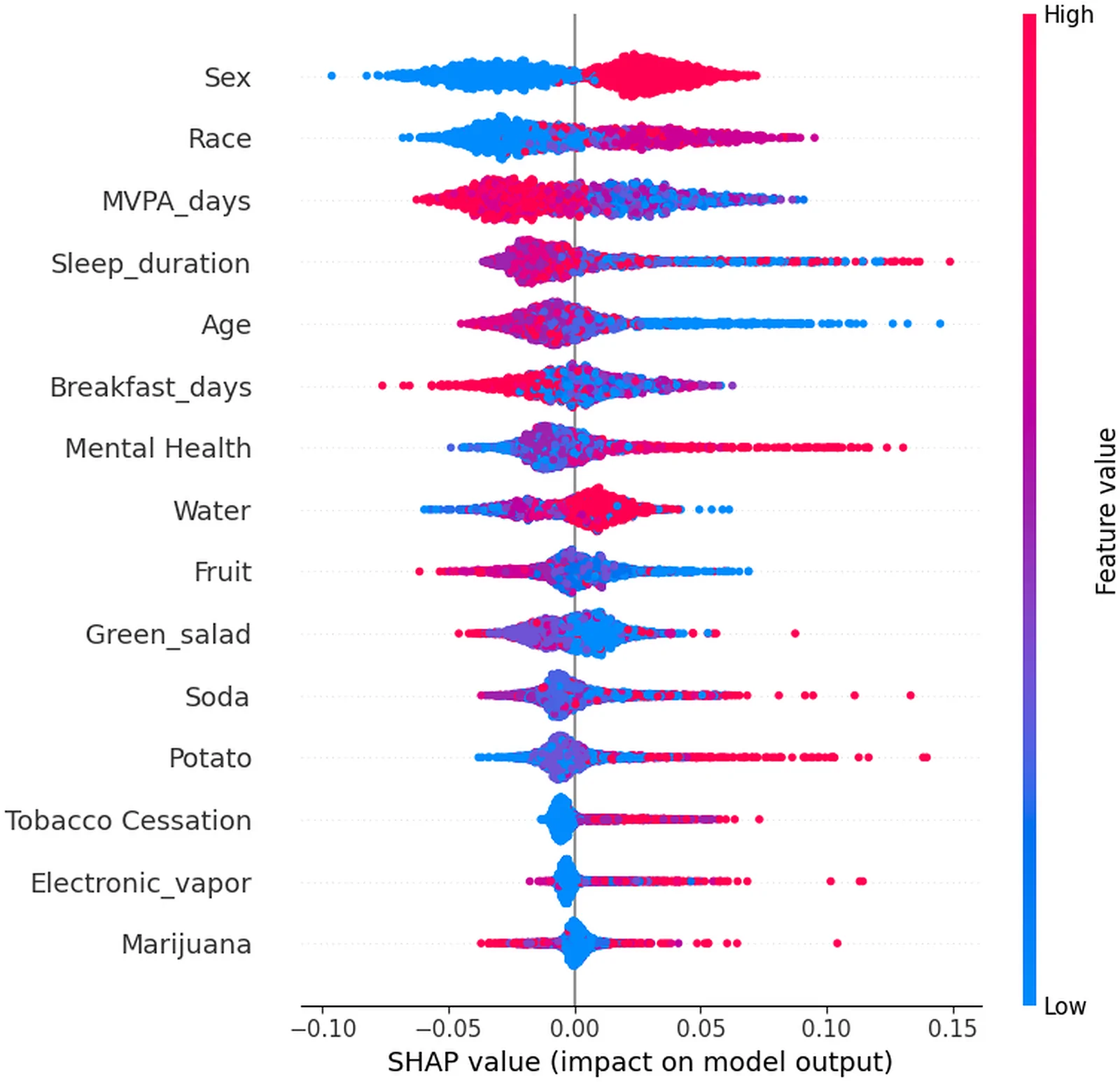
Exploratory SHAP summary plot of RF-Extended model output in the held-out test set. Features are ordered by their overall contribution to model output. Positive and negative SHAP values indicate shifts in the fitted model output and should not be interpreted as causal effects.

## Discussion

### Principal findings

This study shows that adolescent overweight/obesity in YRBSS 2023 reflects both background demographic differences and modifiable behaviors, with an overall overweight/obesity burden of 31.8% in the analytic sample. This finding is consistent with the recent review by Kelly et al., which describes adolescent obesity as a multifactorial condition [6]. In survey-aware analysis, core correlates such as sex, race, MVPA and breakfast were generally consistent across the base and extended models, indicating that expanding the predictor framework did not alter the basic epidemiologic logic of the findings. The central finding of the study is that, in survey-based adolescent public health data, enriching the predictor framework with behavioral and psychosocial variables available in YRBSS provided more consistent benefit for cross-sectional classification of current overweight/obesity status than simply switching between two familiar tree-based algorithms. RF-Extended achieved an AUC of 0.6894 and performed clearly better than RF-Base, while isotonic recalibration largely preserved discrimination and substantially improved probability reliability. The imbalance-handling sensitivity analysis further showed that the main comparison between the extended models was not driven solely by SMOTENC: removing oversampling produced modest reductions in ROC-AUC, sensitivity, and weighted F1 in both RF-Extended and XGB-Extended, while RF-Extended retained higher discrimination. Thus, SMOTENC improved positive-class detection to some extent but did not alter the relative ranking of the two extended models. At the same time, the exploratory SHAP analysis showed that the features contributing most strongly to RF-Extended predictions included several variables with established epidemiologic relevance, including sex, race/ethnicity, physical activity, sleep duration, age and breakfast frequency. Overall, these findings support calibrated machine-learning classification as a transparent way to characterize current overweight/obesity status; any future screening or prioritization use would require prospective and external validation.

### Potential public health relevance and future applications

These findings may inform future research on supportive school-health or community applications, but the present cross-sectional analysis does not establish prospective screening utility. Before any implementation could be considered, the model would require prospective and external validation, together with assessments of feasibility, acceptability, fairness, privacy, and data governance. These considerations are particularly important because the extended model includes sensitive information related to mental health and substance use. The potential consequences of false-positive and false-negative classifications would also need to be evaluated in the intended setting. Any future application should therefore remain supplementary to professional assessment rather than function as an automated decision-making system [30]. The contribution of MVPA, breakfast and sleep to model predictions is consistent with the broader public-health relevance of these behaviors reported in previous literature [31,32]. However, their prominence in the SHAP analysis should not itself be interpreted as evidence that intervention on these variables would change overweight/obesity status. More importantly, the results suggest that enriching the predictor framework with broader behavioral and psychosocial information can improve cross-sectional classification of current overweight/obesity status while preserving the model’s interpretability [9]. Because YRBSS does not fully capture upstream structural determinants, these findings should be interpreted as evidence on the added value of richer individual-level variables rather than as a complete account of social determinants.

### Calibration, equity and responsible use in public health

The calibration results emphasize that discrimination alone is not enough if a model is expected to support public health action. In this study, RF-Extended showed a more favorable calibration profile because after isotonic recalibration, probability ranking was largely preserved while probability reliability improved clearly. Together with the exploratory SHAP analysis showing that the model relied substantially on interpretable demographic, behavioral and psychosocial features, these findings improve transparency regarding how RF-Extended generated its outputs. This interpretability should nevertheless be understood as model explanation rather than evidence of causal or intervention effects. However, responsible AI in public health requires more than calibration and interpretability. It also requires checking whether performance is reasonably consistent across population subgroups. In the subgroup analysis, model discrimination was not fully uniform across sex and race groups. This is not enough to conclude that the model is unfair, but it does show that the model outputs should not be assumed to have the same reliability in every subgroup. Therefore, any future practical evaluation should retain human oversight, subgroup performance checks and context-appropriate interpretation.

### Comparison with previous literature

The current findings are broadly consistent with two lines of previous literature including research on correlates of adolescent overweight/obesity and research on machine-learning classification of overweight/obesity. Regarding correlates, the systematic review by Monzani et al. showed that skipping breakfast is a fairly consistent marker of overweight/obesity risk and adverse cardiometabolic outcomes in children and adolescents [33]. Similarly, the systematic review by Gale et al., which synthesized 89 studies, found that multiple unfavorable sleep dimensions, especially poor sleep hygiene, later sleep timing, high variability and sleep disturbance, were consistently associated with increased obesity and adiposity in adolescents [11]. These findings provide epidemiologic context for why breakfast, MVPA and sleep were prominent in both the survey-aware regression results and the RF-Extended model explanations. However, the two analyses answer different questions: the regression estimates adjusted cross-sectional associations, whereas SHAP describes contributions to fitted-model predictions.

However, the distinctive contribution of this study is that it does not stop at association analysis or model explanation in isolation. Instead, it integrates YRBSS 2023, a broader dfactor framework, survey-aware regression, cross-sectional machine-learning classification, calibration and interpretation within a single public health narrative. This approach evaluates AI as a calibrated and interpretable method for classifying current overweight/obesity status rather than as a prospective prediction system.

### Strengths and limitations

This study has several notable strengths. First, the analysis used YRBSS 2023, a nationally relevant and recent survey dataset that is well suited to the context of adolescent public health. Second, the direct comparison between the base and extended frameworks made it possible to quantify the added value of predictor enrichment more clearly. Third, the study did not stop at regression or machine learning alone but integrated survey-aware regression and cross-sectional machine-learning classification within a single analytic narrative. Fourth, calibration, interpretation and subgroup checking were placed at the center rather than reporting AUC alone, which is a meaningful strength given that recent reviews of obesity prediction still show that calibration is often overlooked.

However, the limitations should also be recognized clearly. First, this was a cross-sectional analysis based on self-reported survey data, so it does not support causal inference and should not be interpreted as prediction of future events. The SHAP analysis was exploratory and was intended only to describe the behavior of the fitted RF-Extended model on held-out observations; SHAP values do not identify independent determinants and cannot support causal inference. In addition, 11.4% of the original sample was excluded because BMI percentile was unavailable, and excluded respondents differed from the included analytic sample in several basic demographic characteristics. Selection bias related to missing outcome data therefore cannot be completely excluded. Missingness was also substantial for several behavioral predictors. However, the complete-case sensitivity analysis produced broadly similar model performance to the primary imputed analysis, suggesting that the principal machine-learning findings were not materially driven by the predictor-imputation procedure. Second, the machine learning component was evaluated only through internal validation within the same survey cycle, so generalizability to other survey years or other population contexts still needs further assessment. Third, although the extended framework enriched individual-level behavioral and psychosocial information, YRBSS still does not measure many upstream structural determinants that may be important for adolescent overweight/obesity. Therefore, model outputs should be understood as estimates for cross-sectional classification of current overweight/obesity status. Prospective and external validation, together with equity assessment, would be required before any wider screening or prioritization use is considered.

## Conclusions

This study shows that expanding the predictor framework with behavioral and psychosocial variables available in YRBSS can improve calibrated cross-sectional classification of current adolescent overweight/obesity status more consistently than changing the algorithm alone. At the same time, the features contributing most strongly to model output included several variables with established public-health relevance, including physical activity, breakfast frequency and sleep duration. The selected model is therefore best understood as a cross-sectional classification model rather than a tool for predicting future obesity. Future studies should prioritize prospective and external validation, followed by evaluation of potential practical use, before any screening or implementation is considered.

## Supporting information

S1 Table Summary of variables investigated in the study and machine-learning encoding. (PDF)

S2 Table Weighted characteristics of participants across weight-status groups. (PDF)

S3 Table Survey-aware multivariable logistic regression of overweight/obesity for the base frameworks. (PDF)

S4 Table Survey-aware multivariable logistic regression of overweight/obesity for the extended frameworks. (PDF)

S5 Table Pairwise comparisons of model performance on the held-out test set. (PDF)

S6 Table Subgroup performance of RF-Extended model across sex and race groups. (PDF)

S7 Table Calibration metrics of the selected final model (RF-Extended) before and after isotonic recalibration. (PDF)

S8 Table Hyperparameter optimization settings and selected parameters. (PDF)

S9 Table Internal stability of model performance using repeated stratified cross-validation. (PDF)

S10 Table RF-Extended operating thresholds and test-set performance at accuracy-oriented and sensitivity-oriented operating points. (PDF)

S11 Table Demographic characteristics of respondents included in and excluded from the analytic sample because of missing BMI-for-age percentile. (PDF)

S12 Table Missing observations for the 16 RF-Extended predictors before imputation. (PDF)

S13 Table Sensitivity analysis comparing the primary imputed and complete-case RF-Extended analyses. (PDF)

S14 Table Sensitivity analysis comparing SMOTENC with no oversampling in the extended machine-learning models. (PDF)

S15 Fig Learning curves for the four machine-learning model configurations. Training and cross-validation weighted F1 scores are shown for RF-Base, RF-Extended, XGB-Base, and XGB-Extended. Weighted F1 was used because it was the optimization objective during hyperparameter tuning. Training performance was generally higher than cross-validation performance, particularly at smaller training sizes, while the gap narrowed as training size increased. Cross-validation weighted F1 approached a plateau in all four models, with the extended models showing higher validation performance than the corresponding base models.
(PNG)

S16 Table Parallel training- and test-set performance and absolute confusion-matrix counts. (PDF)

S17 Fig Preprocessing and machine-learning development pipeline. Overview of the analytical workflow. The BMI-eligible analytic sample was divided into development/training and held-out test sets using a stratified 70/30 split. Within the development data, training-derived imputation, five-fold cross-validation with Optuna tuning, SMOTENC, model-specific encoding, final model fitting, and threshold selection were performed. Isotonic calibration was additionally applied to RF-Extended. The held-out test set was subsequently processed using the frozen preprocessing pipeline and used for final evaluation. Exploratory SHAP analysis was then performed for model interpretation.
(PNG)
