# Correction: Broader factors associated with current adolescent overweight/obesity status in YRBSS 2023: Survey-aware analysis and calibrated AI-assisted classification

**DOI:** 10.1371/journal.pone.0361872

**Published:** 2026-10-30

**Authors:** The PLOS One Staff

There are errors in the author affiliations. The publisher apologizes for the errors.The correct affiliations are as follows:

Thu Hoang Thanh^1^, Nhan Nguyen Thi^2^, Toan Nguyen Khac^3^

**1** University of Economics Ho Chi Minh City–UEH, Ho Chi Minh City, Vietnam, **2** Department of Midwifery, Faculty of Nursing and Medical Technology, University of Medicine and Pharmacy at Ho Chi Minh City, Vietnam, **3** Institute of Intelligent and Interactive Technologies, University of Economics Ho Chi Minh City, Vietnam.
