# Supplementary material for: Broader factors associated with current adolescent overweight/obesity status in YRBSS 2023: Survey-aware analysis and calibrated AI-assisted classification
Source: PLoS One. 2026 Oct 8;21(10):e0360155. doi: 10.1371/journal.pone.0360155 (PMC13649136; doi:10.1371/journal.pone.0360155)
Supplement: Supplementary file 1 [file pone.0360155.s001.pdf]

Supporting information

S1 Table. Summary of variables investigated in the study and machine-learning encoding.

No	Study variable	YRBSS identifiers	Questions asked	Response options provided	Analytic coding and ML encoding
1	Weight status	Calculated variables: BMIPCT, QNOWT, QNOBESE	Derived from self-reported height (Q6) and weight (Q7), with age (Q1) and sex (Q2) incorporated in YRBS edited calculations.	Not directly asked; based on free-text self-reported height (in feet/inches) and weight (in pounds).	4 groups: underweight (<5th), normal weight (5th to <85th), overweight (85th to <95th), and obese (≥95th percentile). Binary analyses used non-overweight vs overweight/obesity. For machine learning, the binary outcome was coded 0 = non-overweight and 1 = overweight/obesity.
2	Age	Q1	How old are you?	A. 12 years old or younger<br>B. 13 years old<br>C. 14 years old<br>D. 15 years old<br>E. 16 years old<br>F. 17 years old<br>G. 18 years old or older	A. 14 years old or younger<br>B. 15 years old<br>C. 16 years old<br>D. 17 years old<br>E. 18 years old or older<br>For machine learning: ordinal encoding in ascending age order.
3	Sex	Q2	What is your sex?	A. Male<br>B. Female	A. Male<br>B. Female<br>For machine learning: binary indicator, Female = 0 and Male = 1.
4	Race	Calculated Variable: raceeth	What is your race?<br><br>Are you Hispanic or Latino?	A. American Indian or Alaska Native<br>B. Asian<br>C. Black or African American<br>D. Native Hawaiian or Other Pacific Islander<br>E. White<br>A. Yes<br>B. No	A. White<br>B. Black or African American<br>C. Hispanic or Latino<br>D. All Other Races<br>Nominal one-hot encoding across the four race/ethnicity categories; no ordinal ordering was imposed.
5	MVPA	Q77	During the past 7 days, on how many days were you physically active for a total of at least 60 minutes per day? (Add up all the time you spent in any kind of physical activity that increased your heart rate and made you breathe hard some of the time.)	A. 0 days<br>B. 1 day<br>C. 2 days<br>D. 3 days<br>E. 4 days<br>F. 5 days<br>G. 6 days<br>H. 7 days	Ordinal encoding from 0 days to 7 days in increasing activity frequency.

No	Study variable	YRBSS identifiers	Questions asked	Response options provided	Analytic coding and ML encoding
6	Sleep	Q86	On an average school night, how many hours of sleep do you get?	A. 4 hours or less<br>B. 5 hours<br>C. 6 hours<br>D. 7 hours<br>E. 8 hours<br>F. 9 hours<br>G. 10 or more hours	Ordinal encoding in increasing sleep-duration order.
7	Breakfast days	Q76	During the past 7 days, how many days did you eat breakfast?	A. 0 days<br>B. 1 day<br>C. 2 days<br>D. 3 days<br>E. 4 days<br>F. 5 days<br>G. 6 days<br>H. 7 days	Ordinal encoding from 0 days to 7 days in increasing breakfast frequency.
8	Potatoes	Q71	During the past 7 days, how many times did you eat potatoes? (Do not count french fries, fried potatoes, or potato chips.)	A. Did not eat potatoes<br>B. 1 to 3 times<br>C. 4 to 6 times<br>D. 1 time per day<br>E. 2 times per day<br>F. 3 times per day<br>G. 4 or more times per day	Ordinal encoding in increasing consumption-frequency order.
9	Green salad	Q70	During the past 7 days, how many times did you eat green salad?	A. Did not eat green salad<br>B. 1 to 3 times<br>C. 4 to 6 times<br>D. 1 time per day<br>E. 2 times per day<br>F. 3 times per day<br>G. 4 or more times per day	Ordinal encoding in increasing consumption-frequency order.
10	Soda	Q74	During the past 7 days, how many times did you drink a can, bottle, or glass of soda or pop, such as Coke, Pepsi, or Sprite? (Do not count diet soda or diet Pop.)	A. Did not eat/drink<br>B. 1 to 3 times<br>C. 4 to 6 times<br>D. 1 time per day<br>E. 2 times per day<br>F. 3 times per day<br>G. 4 or more times per day	Ordinal encoding in increasing consumption-frequency order.
11	Fruit juice	Q68	During the past 7 days, how many times did you drink 100% fruit juices such as orange juice, apple juice, or grape juice? (Do not count punch, KoolAid, sports drinks, or other fruit-flavored drinks.)	A. Did not eat/drink<br>B. 1 to 3 times<br>C. 4 to 6 times<br>D. 1 time per day<br>E. 2 times per day<br>F. 3 times per day<br>G. 4 or more times per day	Ordinal encoding in increasing consumption-frequency order.
12	Water	Q96	During the past 7 days, how many times did you drink a bottle or glass of plain water?	A. I did not drink water during the past 7 days<br>B. 1 to 3 times during the past 7 days<br>C. 4 to 6 times during the past 7 days<br>D. 1 time per day<br>E. 2 times per day<br>F. 3 times per day<br>G. 4 or more times per day	Ordinal encoding in increasing consumption-frequency order.

No	Study variable	YRBSS identifiers	Questions asked	Response options provided	Analytic coding and ML encoding
13	Mental health	Q84	During the past 30 days, how often was your mental health not good? (Poor mental health includes stress, anxiety, and depression.)	A. Always<br>B. Most of the time<br>C. Never<br>D. Rarely<br>E. Sometimes	Ordinal encoding in the order Never < Rarely < Sometimes < Most of the time < Always.
14	Electronic vapor	Q36	During the past 30 days, on how many days did you use an electronic vapor product?	A. 0 days<br>B. 1 or 2 days<br>C. 3 to 5 days<br>D. 6 to 9 days<br>E. 10 to 19 days<br>F. 20 to 29 days<br>G. All 30 days	Ordinal encoding in increasing electronic-vapor-use frequency.
15	Alcohol	Q42	During the past 30 days, on how many days did you have at least one drink of alcohol?	A. 0 days<br>B. 1 or 2 days<br>C. 3 to 5 days<br>D. 6 to 9 days<br>E. 10 to 19 days<br>F. 20 to 29 days	Ordinal encoding in increasing alcohol-use frequency.
16	All tobacco product cessation	Q40	During the past 12 months, did you ever try to quit using all tobacco products?	A. I did not use cigarettes, electronic vapor products, smokeless tobacco, cigars, shisha or hookah tobacco, pipe tobacco, heated tobacco products, or nicotine pouches during the past 12 months<br>B. Yes<br>C. No	Nominal one-hot encoding across the three response categories; no ordinal ordering was imposed.
17	Marijuana	Q46	During your life, how many times have you used marijuana?	A. 0 times<br>B. 1 or 2 times<br>C. 3 to 9 times<br>D. 10 to 19 times<br>E. 20 to 39 times<br>F. 40 to 99 times<br>G. 100 or more times	Ordinal encoding in increasing lifetime marijuana-use frequency.

Note. Ordinal predictors were encoded according to their natural category order. Race/ethnicity and tobacco-cessation status were treated as nominal variables and one-hot encoded, and sex was represented as a binary indicator. One-hot encoding was used specifically to avoid imposing an artificial ordering on nominal categories.
