# Supplementary material for: Broader factors associated with current adolescent overweight/obesity status in YRBSS 2023: Survey-aware analysis and calibrated AI-assisted classification
Source: PLoS One. 2026 Oct 8;21(10):e0360155. doi: 10.1371/journal.pone.0360155 (PMC13649136; doi:10.1371/journal.pone.0360155)
Supplement: Supplementary file 2 [file pone.0360155.s002.pdf]

Supporting information

S2 Table. Weighted characteristics of participants across weight-status groups.

Variable	Non-overweight (n=12145)	Overweight (n=2710)	Obese (n=2959)	X^2	P value
**Sex**					
Female	6133 (48.0%)	1360 (48.2%)	1158 (39.1%)	76.839	<0.001
Male	6012 (52.0%)	1350 (51.8%)	1801 (60.9%)		
**Age**					
14 years old or younger	1502 (11.2%)	360 (12.8%)	379 (14.4%)	38.824	0.091
15 years old	3390 (25.6%)	739 (25.7%)	760 (23.2%)		
16 years old	3217 (26.2%)	712 (27.4%)	730 (24.7%)		
17 years old	2678 (24.8%)	628 (22.6%)	737 (25.9%)		
18 years old or older	1358 (12.2%)	271 (11.5%)	353 (11.7%)		
**Race**					
White	6462 (51.7%)	1274 (45.0%)	1254 (41.3%)	189.996	<0.001
Black or African American	1021 (12.1%)	272 (16.1%)	332 (17.2%)		
Hispanic/Latino	3254 (30.4%)	828 (29.3%)	966 (32.7%)		
All Other Races	1408 (5.8%)	336 (9.6%)	407 (8.7%)		
**MVPA days**					
0 days	1567 (13.7%)	380 (16.0%)	502 (18.8%)	168.812	<0.001
1 day	751 (6.5%)	177 (7.0%)	211 (7.1%)		
2 days	972 (9.4%)	239 (8.6%)	305 (11.4%)		
3 days	1198 (10.7%)	300 (11.4%)	357 (11.6%)		
4 days	1113 (9.5%)	274 (10.2%)	310 (12.0%)		
5 days	1689 (14.1%)	362 (13.5%)	415 (14.9%)		
6 days	1055 (8.8%)	231 (9.3%)	160 (5.8%)		
7 days	3800 (27.3%)	747 (23.9%)	699 (18.4%)		
**Breakfast days**					
0 days	1532 (15.7%)	445 (20.7%)	531 (22.9%)	179.312	<0.001
1 day	780 (8.9%)	206 (10.0%)	210 (10.4%)		
2 days	1075 (13.2%)	277 (14.8%)	340 (13.5%)		
3 days	947 (10.6%)	249 (11.3%)	309 (13.9%)		
4 days	694 (8.5%)	149 (7.9%)	153 (5.8%)		
5 days	708 (7.7%)	151 (8.7%)	191 (8.0%)		
6 days	505 (5.9%)	85 (4.5%)	93 (4.4%)		
7 days	2312 (29.4%)	410 (22.0%)	430 (21.0%)		
**Sleep**					
4 hours or less	867 (8.3%)	215 (6.7%)	329 (10.5%)	71.726	0.028
5 hours	1501 (14.0%)	347 (14.1%)	430 (15.6%)		
6 hours	2677 (25.0%)	613 (27.6%)	677 (27.1%)		
7 hours	3114 (29.7%)	670 (30.2%)	670 (27.4%)		
8 hours	1878 (17.9%)	379 (15.1%)	416 (15.0%)		
9 hours	441 (4.0%)	97 (4.5%)	88 (2.8%)		
10 hours or more	122 (1.2%)	47 (1.9%)	35 (1.7%)		
**Potatoes**					
Did not consume	3452 (37.6%)	790 (39.3%)	800 (36.8%)	74.962	0.007
1 to 3 times	4400 (47.1%)	923 (42.0%)	1068 (46.7%)		
4 to 6 times	868 (8.6%)	243 (10.4%)	249 (8.1%)		
1 time per day	354 (3.9%)	85 (3.7%)	98 (3.4%)		
2 times per day	145 (1.4%)	56 (3.2%)	54 (2.6%)		
3 times per day	71 (0.7%)	13 (0.4%)	19 (0.7%)		
4 times per day	110 (0.8%)	22 (0.9%)	37 (1.6%)		
**Fruit juice**					
Did not consume	1310 (10.5%)	305 (11.3%)	361 (13.4%)	64.869	0.046
1 to 3 times	3921 (32.9%)	921 (35.6%)	976 (32.2%)		
4 to 6 times	2487 (22.7%)	586 (23.4%)	603 (22.3%)		
1 time per day	1365 (12.1%)	275 (10.4%)	293 (10.3%)		
2 times per day	1418 (12.5%)	291 (11.3%)	314 (11.1%)		
3 times per day	532 (4.8%)	97 (2.8%)	129 (5.2%)		
4 times per day	542 (4.5%)	128 (5.2%)	166 (5.4%)		
**Green salad**					
Did not consume	4184 (42.7%)	939 (44.7%)	1068 (44.7%)	42.817	0.132
1 to 3 times	3553 (39.1%)	799 (35.8%)	824 (36.1%)		
4 to 6 times	795 (8.8%)	180 (9.8%)	189 (9.3%)		

Variable	Non-overweight (n=12145)	Overweight (n=2710)	Obese (n=2959)	X^2	P value
1 time per day	523 (5.4%)	123 (5.3%)	137 (5.8%)		
2 times per day	194 (2.3%)	45 (1.5%)	55 (2.0%)		
3 times per day	59 (0.8%)	17 (1.2%)	16 (0.3%)		
4 times per day	103 (1.0%)	27 (1.5%)	41 (1.8%)		
**Soda**					
Did not consume	2739 (31.1%)	633 (31.0%)	575 (28.4%)	32.298	0.275
1 to 3 times	3702 (40.9%)	826 (39.8%)	901 (40.9%)		
4 to 6 times	1359 (14.0%)	311 (15.3%)	386 (14.8%)		
1 time per day	691 (7.1%)	157 (7.1%)	171 (6.3%)		
2 times per day	427 (3.7%)	100 (4.0%)	135 (5.3%)		
3 times per day	168 (1.3%)	36 (1.5%)	52 (1.6%)		
4 times per day	226 (2.0%)	49 (1.3%)	91 (2.7%)		
**Electronic vapor**					
Did not use	9565 (83.0%)	2084 (82.7%)	2281 (83.3%)	39.976	0.86
1 to 2 days	480 (4.7%)	143 (4.1%)	139 (4.9%)		
3 to 5 days	226 (2.0%)	73 (3.7%)	79 (2.8%)		
6 to 9 days	178 (1.5%)	41 (1.8%)	43 (1.5%)		
10 to 19 days	252 (2.2%)	55 (1.6%)	65 (1.8%)		
20 to 29 days	202 (1.6%)	38 (1.2%)	39 (1.1%)		
All 30 days	676 (5.0%)	160 (4.9%)	168 (4.5%)		
**Alcohol**					
Did not use	9083 (76.7%)	2039 (79.4%)	2239 (79.3%)	58.574	0.061
1 to 2 days	1488 (13.6%)	318 (12.2%)	359 (13.2%)		
3 to 5 days	578 (5.3%)	119 (4.1%)	121 (3.9%)		
6 to 9 days	283 (2.2%)	64 (2.4%)	52 (2.0%)		
10 to 19 days	161 (1.5%)	33 (1.4%)	33 (0.6%)		
20 to 29 days	37 (0.3%)	13 (0.6%)	10 (0.3%)		
All 30 days	40 (0.3%)	4 (0.0%)	22 (0.8%)		
**Marijuana**					
Did not use	6805 (69.7%)	1518 (69.2%)	1692 (70.1%)	25.846	0.545
1 to 2 days	742 (7.1%)	208 (9.2%)	207 (7.9%)		
3 to 5 days	616 (6.3%)	154 (5.7%)	164 (6.0%)		
6 to 9 days	357 (4.1%)	92 (3.2%)	96 (3.3%)		
10 to 19 days	285 (3.2%)	65 (3.5%)	81 (3.0%)		
20 to 29 days	252 (2.4%)	70 (2.8%)	83 (3.0%)		
All 30 days	870 (7.3%)	188 (6.5%)	217 (6.6%)		
**Mental health**					
Never	1730 (18.3%)	370 (19.5%)	405 (18.0%)	42.175	0.055
Rarely	2115 (23.0%)	442 (19.0%)	512 (22.4%)		
Sometimes	2959 (31.1%)	647 (31.5%)	677 (27.8%)		
Most of the time	2090 (20.1%)	478 (22.1%)	531 (21.7%)		
Always	803 (7.5%)	203 (7.9%)	242 (10.1%)		
**All tobacco product cessation**					
Did not use tobacco products	6608 (72.9%)	1478 (71.1%)	1635 (72.6%)	22.353	0.078
Yes	1390 (13.4%)	348 (14.6%)	411 (16.3%)		
No	1167 (13.6%)	306 (14.3%)	303 (11.1%)		
**Water intake**					
Did not consume	251 (2.7%)	41 (2.2%)	68 (3.0%)	74.613	0.004
1 to 3 times	710 (7.3%)	164 (7.4%)	203 (9.0%)		
4 to 6 times	1010 (11.2%)	212 (11.2%)	264 (12.6%)		
1 time per day	737 (8.4%)	135 (7.0%)	133 (5.6%)		
2 times per day	1334 (17.8%)	293 (16.0%)	288 (14.0%)		
3 times per day	1297 (17.1%)	314 (15.9%)	323 (13.8%)		
4 times per day	2819 (35.6%)	725 (40.2%)	865 (42.0%)		
