# Supplementary material for: Broader factors associated with current adolescent overweight/obesity status in YRBSS 2023: Survey-aware analysis and calibrated AI-assisted classification
Source: PLoS One. 2026 Oct 8;21(10):e0360155. doi: 10.1371/journal.pone.0360155 (PMC13649136; doi:10.1371/journal.pone.0360155)
Supplement: Supplementary file 3 [file pone.0360155.s003.pdf]

Supporting information

S3 Table. Survey-aware multivariable logistic regression of overweight/obesity for the base frameworks.

Variables	Odds Ratio	95%CI	p
**Age** (ref: 14 years old or younger)			
15 years old	0.76	0.60–0.96	0.022
16 years old	0.84	0.67–1.06	0.147
17 years old	0.78	0.61–0.99	0.044
18 years old or older	0.81	0.60–1.11	0.185
**Sex** (ref: Female)			
Male	1.33	1.16–1.52	< 0.001
**Race** (ref: White)			
Black or African American	1.51	1.20–1.88	0.001
Hispanic/Latino	1.81	1.35–2.41	< 0.001
All Other Races	1.12	0.93–1.34	0.226
**MVPA days** (ref: 0 days)			
1 day	0.90	0.67–1.21	0.468
2 days	0.90	0.64–1.27	0.543
3 days	0.97	0.78–1.20	0.754
4 days	0.97	0.73–1.29	0.819
5 days	0.90	0.71–1.13	0.349
6 days	0.91	0.60–1.38	0.656
7 days	0.70	0.55–0.90	0.005
**Sleep** (ref: 4 hours or less)			
5 hours	1.02	0.78–1.32	0.891
6 hours	1.16	0.95–1.43	0.144
7 hours	1.02	0.78–1.32	0.908
8 hours	0.96	0.76–1.22	0.728
9 hours	0.96	0.69–1.33	0.784
10 hours or more	1.28	0.67–2.42	0.447
**Breakfast days** (ref: 0 days)			
1 day	0.82	0.59–1.13	0.214
2 days	0.87	0.69–1.10	0.247
3 days	0.95	0.73–1.22	0.653
4 days	0.58	0.47–0.71	< 0.001
5 days	0.79	0.65–0.96	0.017
6 days	0.65	0.48–0.88	0.007
7 days	0.59	0.48–0.72	< 0.001
**Potatoes** (ref: Did not consume)			
1 to 3 times	1.01	0.87–1.18	0.863
4 to 6 times	1.20	0.94–1.52	0.143
1 time per day	0.78	0.56–1.10	0.153
2 times per day	1.90	1.12–3.20	0.017
3 times per day	0.51	0.21–1.23	0.131
4 or more times per day	1.53	0.85–2.76	0.154
**Fruit juice** (ref: Did not consume)			
1 to 3 times	0.92	0.75–1.13	0.404
4 to 6 times	0.90	0.72–1.12	0.329
1 time per day	0.80	0.59–1.07	0.132
2 times per day	0.93	0.71–1.21	0.584
3 times per day	0.72	0.45–1.16	0.175
4 or more times per day	0.95	0.57–1.57	0.828
**Green salad** (ref: Did not consume)			

**Variables**	**Odds Ratio**	**95%CI**	**p**
1 to 3 times	0.99	0.86–1.14	0.901
4 to 6 times	1.16	0.91–1.48	0.236
1 time per day	1.28	0.96–1.72	0.094
2 times per day	1.06	0.69–1.63	0.785
3 times per day	1.68	0.74–3.83	0.215
4 or more times per day	1.50	0.89–2.53	0.124
**Soda** (ref: Did not consume)			
1 to 3 times	1.10	0.95–1.26	0.199
4 to 6 times	1.02	0.81–1.28	0.892
1 time per day	1.04	0.75–1.45	0.816
2 times per day	1.31	0.95–1.81	0.100
3 times per day	1.00	0.56–1.77	0.985
4 or more times per day	0.74	0.46–1.17	0.189
**Alcohol** (ref: Did not use)			
1 to 2 days	0.90	0.77–1.07	0.222
3 to 5 days	0.74	0.52–1.05	0.090
6 to 9 days	0.80	0.45–1.41	0.431
10 to 19 days	0.66	0.31–1.42	0.281
20 to 29 days	0.35	0.12–1.06	0.063
All 30 days	1.11	0.31–3.98	0.874
**Current electronic vapor** (ref: Did not use)			
1 to 2 days	0.98	0.71–1.37	0.914
3 to 5 days	1.47	0.93–2.34	0.098
6 to 9 days	1.22	0.84–1.78	0.296
10 to 19 days	0.88	0.55–1.41	0.591
20 to 29 days	0.80	0.47–1.35	0.395
All 30 days	0.97	0.68–1.38	0.847
**Marijuana** (ref: Did not use)			
1 to 2 times	1.17	0.90–1.52	0.242
3 to 9 times	0.95	0.72–1.26	0.739
10 to 19 times	0.75	0.55–1.02	0.069
20 to 39 times	1.03	0.67–1.57	0.905
40 to 99 times	1.42	1.08–1.87	0.013
100 or more times	1.01	0.78–1.31	0.924
