# Supplementary material for: Broader factors associated with current adolescent overweight/obesity status in YRBSS 2023: Survey-aware analysis and calibrated AI-assisted classification
Source: PLoS One. 2026 Oct 8;21(10):e0360155. doi: 10.1371/journal.pone.0360155 (PMC13649136; doi:10.1371/journal.pone.0360155)
Supplement: Supplementary file 4 [file pone.0360155.s004.pdf]

# Supporting information

**S4 Table. Survey-aware multivariable logistic regression of overweight/obesity for the extended frameworks.**

| Variables | Odds Ratio | 95%CI | p |
|---|---|---|---|
| **Age** (ref: 14 years old or younger) | | | |
| 15 years old | 0.74 | 0.57–0.96 | 0.024 |
| 16 years old | 0.85 | 0.67–1.08 | 0.19 |
| 17 years old | 0.76 | 0.59–0.98 | 0.033 |
| 18 years old or older | 0.82 | 0.59–1.14 | 0.222 |
| **Sex** (ref: Female) | | | |
| Male | 1.34 | 1.17–1.53 | <0.001 |
| **Race** (ref: White) | | | |
| Black or African American | 1.51 | 1.18–1.93 | 0.001 |
| Hispanic/Latino | 1.95 | 1.44–2.65 | <0.001 |
| All Other Races | 1.07 | 0.91–1.27 | 0.396 |
| **MVPA days** (ref: 0 days) | | | |
| 1 day | 0.89 | 0.65–1.22 | 0.465 |
| 2 days | 0.91 | 0.63–1.31 | 0.602 |
| 3 days | 0.94 | 0.74–1.20 | 0.635 |
| 4 days | 0.96 | 0.70–1.33 | 0.82 |
| 5 days | 0.86 | 0.67–1.11 | 0.25 |
| 6 days | 0.89 | 0.57–1.38 | 0.603 |
| 7 days | 0.67 | 0.52–0.87 | 0.003 |
| **Sleep** (ref: 4 hours or less) | | | |
| 5 hours | 1.07 | 0.81–1.41 | 0.644 |
| 6 hours | 1.32 | 1.06–1.63 | 0.013 |
| 7 hours | 1.17 | 0.89–1.54 | 0.254 |
| 8 hours | 1.07 | 0.83–1.38 | 0.598 |
| 9 hours | 1.12 | 0.81–1.53 | 0.493 |
| 10 hours or more | 1.52 | 0.76–3.06 | 0.23 |
| **Breakfast days** (ref: 0 days) | | | |
| 1 day | 0.85 | 0.60–1.19 | 0.327 |
| 2 days | 0.84 | 0.64–1.12 | 0.232 |
| 3 days | 0.97 | 0.74–1.26 | 0.804 |
| 4 days | 0.60 | 0.49–0.73 | <0.001 |
| 5 days | 0.79 | 0.65–0.97 | 0.023 |
| 6 days | 0.68 | 0.49–0.93 | 0.016 |
| 7 days | 0.59 | 0.47–0.75 | <0.001 |
| **Potatoes** | | | |
| (ref: Did not consume) | | | |
| 1 to 3 times | 1.02 | 0.87–1.19 | 0.842 |
| 4 to 6 times | 1.19 | 0.94–1.52 | 0.153 |
| 1 time per day | 0.76 | 0.53–1.08 | 0.123 |
| 2 times per day | 2.13 | 1.23–3.72 | 0.008 |
| 3 times per day | 0.47 | 0.18–1.22 | 0.12 |
| 4 or more times per day | 1.46 | 0.82–2.63 | 0.197 |
| **Fruit juice** (ref: Did not consume) | | | |
| 1 to 3 times | 0.96 | 0.78–1.19 | 0.725 |
| 4 to 6 times | 0.95 | 0.74–1.21 | 0.644 |
| 1 time per day | 0.85 | 0.62–1.18 | 0.328 |
| 2 times per day | 0.94 | 0.71–1.25 | 0.668 |
| 3 times per day | 0.68 | 0.40–1.17 | 0.160 |
| 4 or more times per day | 0.91 | 0.54–1.52 | 0.702 |
| **Green salad** | | | |
| (ref: Did not consume) | | | |

| Variables | Odds Ratio | 95%CI | p |
|---|---|---|---|
| 1 to 3 times | 1.00 | 0.87–1.14 | 0.981 |
| 4 to 6 times | 1.08 | 0.85–1.37 | 0.526 |
| 1 time per day | 1.22 | 0.88–1.69 | 0.236 |
| 2 times per day | 1.02 | 0.62–1.69 | 0.931 |
| 3 times per day | 1.49 | 0.64–3.48 | 0.348 |
| 4 or more times per day | 1.39 | 0.81–2.40 | 0.232 |
| **Soda** (ref: Did not consume) | | | |
| 1 to 3 times | 1.15 | 0.99–1.34 | 0.059 |
| 4 to 6 times | 1.07 | 0.83–1.37 | 0.591 |
| 1 time per day | 1.17 | 0.85–1.59 | 0.331 |
| 2 times per day | 1.39 | 1.00–1.92 | 0.047 |
| 3 times per day | 1.12 | 0.63–2.00 | 0.693 |
| 4 or more times per day | 0.69 | 0.44–1.08 | 0.1 |
| **Alcohol** (ref: Did not use) | | | |
| 1 to 2 days | 0.91 | 0.78–1.07 | 0.253 |
| 3 to 5 days | 0.69 | 0.47–1.02 | 0.062 |
| 6 to 9 days | 0.78 | 0.44–1.39 | 0.391 |
| 10 to 19 days | 0.57 | 0.27–1.20 | 0.134 |
| 20 to 29 days | 0.34 | 0.12–0.97 | 0.043 |
| All 30 days | 1.17 | 0.34–4.02 | 0.8 |
| **Current electronic vapor** (ref: Did not use) | | | |
| 1 to 2 days | 0.86 | 0.60–1.25 | 0.432 |
| 3 to 5 days | 1.28 | 0.71–2.30 | 0.405 |
| 6 to 9 days | 1.08 | 0.70–1.65 | 0.726 |
| 10 to 19 days | 0.76 | 0.44–1.33 | 0.335 |
| 20 to 29 days | 0.65 | 0.35–1.21 | 0.171 |
| All 30 days | 0.92 | 0.58–1.44 | 0.699 |
| **Marijuana** (ref: Did not use) | | | |
| 1 to 2 times | 1.06 | 0.78–1.43 | 0.721 |
| 3 to 9 times | 0.92 | 0.66–1.28 | 0.614 |
| 10 to 19 times | 0.68 | 0.48–0.96 | 0.029 |
| 20 to 39 times | 1.01 | 0.64–1.60 | 0.97 |
| 40 to 99 times | 1.34 | 0.96–1.86 | 0.082 |
| 100 or more times | 0.90 | 0.70–1.14 | 0.372 |
| **Mental health** (ref: Never) | | | |
| Rarely | 0.84 | 0.67–1.06 | 0.146 |
| Sometimes | 0.87 | 0.71–1.06 | 0.160 |
| Most of the time | 1.05 | 0.84–1.32 | 0.672 |
| Always | 1.21 | 0.91–1.60 | 0.178 |
| **All tobacco product cessation** (ref: Did not use tobacco products) | | | |
| Yes | 1.40 | 1.03–1.92 | 0.035 |
| No | 1.03 | 0.77–1.38 | 0.838 |
| **Water intake** (ref: Did not consume) | | | |
| 1 to 3 times | 1.05 | 0.70–1.57 | 0.808 |
| 4 to 6 times | 1.06 | 0.71–1.58 | 0.775 |
| 1 time per day | 0.82 | 0.54–1.26 | 0.364 |
| 2 times per day | 0.92 | 0.63–1.34 | 0.661 |
| 3 times per day | 0.97 | 0.65–1.45 | 0.875 |
| 4 or more times per day | 1.35 | 0.93–1.95 | 0.115 |
