# Supplementary material for: Broader factors associated with current adolescent overweight/obesity status in YRBSS 2023: Survey-aware analysis and calibrated AI-assisted classification
Source: PLoS One. 2026 Oct 8;21(10):e0360155. doi: 10.1371/journal.pone.0360155 (PMC13649136; doi:10.1371/journal.pone.0360155)
Supplement: Supplementary file 5 [file pone.0360155.s005.pdf]

Supporting information

S5 Table. Pairwise comparisons of model performance on the held-out test set.

Comparison	AUC 1	AUC 2	ΔAUC	DeLong p-value	McNemar p-value
RF-Extended vs RF-Base	0.6894	0.6241	+0.0653	<0.001	<0.001
XGB-Extended vs XGB-Base	0.6724	0.6217	+0.0507	<0.001	<0.001
RF-Extended vs XGB-Extended	0.6894	0.6724	+0.0170	0.029	0.170

Note. AUC = area under the receiver operating characteristic curve. ΔAUC was calculated as AUC of the first model minus AUC of the second model. McNemar tests refer to predictions generated at the primary accuracy-oriented operating points.
