# Supplementary material for: Broader factors associated with current adolescent overweight/obesity status in YRBSS 2023: Survey-aware analysis and calibrated AI-assisted classification
Source: PLoS One. 2026 Oct 8;21(10):e0360155. doi: 10.1371/journal.pone.0360155 (PMC13649136; doi:10.1371/journal.pone.0360155)
Supplement: Supplementary file 6 [file pone.0360155.s006.pdf]

Supporting information

S6 Table. Subgroup performance of RF-Extended model across sex and race groups.

Subgroup	AUC	Accuracy	Sensitivity	Specificity	PPV	NPV	Positive-class F1
**All test data**	0.6894	0.6487	0.5640	0.6878	0.4553	0.7732	0.5038
**Female**	0.6711	0.6439	0.5186	0.6942	0.4051	0.7822	0.4549
**Male**	0.6967	0.6512	0.5915	0.6808	0.4790	0.7706	0.5293
**White**	0.6923	0.6743	0.5208	0.7334	0.4293	0.7989	0.4707
**Black or African American**	0.6459	0.5679	0.6211	0.5374	0.4348	0.7123	0.5115
**Hispanic/Latino**	0.6847	0.6132	0.6376	0.6000	0.4627	0.7540	0.5363
**All other races**	0.6612	0.6677	0.4744	0.7606	0.4880	0.7506	0.4811

Note. Subgroup analyses used the same primary accuracy-oriented RF-Extended operating point as the overall test-set analysis. No subgroup-specific threshold optimization was performed.
