# Supplementary material for: Broader factors associated with current adolescent overweight/obesity status in YRBSS 2023: Survey-aware analysis and calibrated AI-assisted classification
Source: PLoS One. 2026 Oct 8;21(10):e0360155. doi: 10.1371/journal.pone.0360155 (PMC13649136; doi:10.1371/journal.pone.0360155)
Supplement: Supplementary file 7 [file pone.0360155.s007.pdf]

Supporting information

S7 Table. Calibration metrics of the selected final model (RF-Extended) before and after isotonic recalibration.

Method	AUC	Brier score	ECE (10 bins)	Calibration intercept	Calibration slope
**Raw RF-Extended**	0.6894	0.212635	0.115980	-0.523191	1.084020
**Isotonic RF-Extended**	0.6891	0.195960	0.021274	-0.085928	0.862979

Note. Isotonic recalibration was based on validation data within the training partition and fixed before evaluation on the held-out test set. For RF-Extended, the accuracy-oriented threshold corresponded to 0.564 before calibration and 0.439 after calibration. The sensitivity-oriented threshold corresponded to 0.428 before calibration and 0.304 after calibration.
