# Supplementary material for: Broader factors associated with current adolescent overweight/obesity status in YRBSS 2023: Survey-aware analysis and calibrated AI-assisted classification
Source: PLoS One. 2026 Oct 8;21(10):e0360155. doi: 10.1371/journal.pone.0360155 (PMC13649136; doi:10.1371/journal.pone.0360155)
Supplement: Supplementary file 8 [file pone.0360155.s008.pdf]

Supporting information

S8 Table. Hyperparameter optimization settings and selected parameters.

Model	Hyperparameter	Search range / candidate values	Selected value
**RF-Base**	n_estimators	50–1000, step 50	50
	max_features	sqrt, log2, 0.5, 0.8	log2
	max_depth	4–24, step 2	20
	min_samples_split	2–20, step 2	6
	min_samples_leaf	1–8	8
**RF-Extended**	n_estimators	50–1000, step 50	700
	max_features	sqrt, log2, 0.5, 0.8	log2
	max_depth	4–24, step 2	24
	min_samples_split	2–20, step 2	12
	min_samples_leaf	1–8	1
**XGB-Base**	n_estimators	50–1000, step 50	550
	max_depth	3–10	7
	learning_rate	0.0050–0.2000, log scale	0.1936
	subsample	0.7000–1.0000	0.8567
	colsample_bytree	0.7000–1.0000	0.9913
	min_child_weight	1–12	7
	gamma	0.0000–5.0000	0.0305
	reg_alpha	0.0001–10.0000, log scale	0.0001
	reg_lambda	0.0010–20.0000, log scale	0.2045
**XGB-Extended**	n_estimators	50–1000, step 50	700
	max_depth	3–10	9
	learning_rate	0.0050–0.2000, log scale	0.1862
	subsample	0.7000–1.0000	0.9367
	colsample_bytree	0.7000–1.0000	0.7894
	min_child_weight	1–12	1
	gamma	0.0000–5.0000	0.1248
	reg_alpha	0.0001–10.0000, log scale	0.0586
	reg_lambda	0.0010–20.0000, log scale	0.2330

Note. RF = Random Forest; XGB = XGBoost. Hyperparameter optimization was performed using Optuna with 500 trials for each model configuration. The weighted F1 score was used as the optimization objective.
