# Supplementary material for: Broader factors associated with current adolescent overweight/obesity status in YRBSS 2023: Survey-aware analysis and calibrated AI-assisted classification
Source: PLoS One. 2026 Oct 8;21(10):e0360155. doi: 10.1371/journal.pone.0360155 (PMC13649136; doi:10.1371/journal.pone.0360155)
Supplement: Supplementary file 9 [file pone.0360155.s009.pdf]

Supporting information

S9 Table. Internal stability of model performance using repeated stratified cross-validation.

Model	ROC-AUC, mean ± SD	Weighted F1, mean ± SD	Positive-class F1, mean ± SD
**RF-Base**	0.6230 ± 0.0130	0.6200 ± 0.0120	0.4590 ± 0.0180
**RF-Extended**	0.6870 ± 0.0120	0.6550 ± 0.0110	0.5010 ± 0.0160
**XGB-Base**	0.6210 ± 0.0140	0.6290 ± 0.0130	0.4710 ± 0.0190
**XGB-Extended**	0.6700 ± 0.0130	0.6540 ± 0.0120	0.4920 ± 0.0170

Note. AUC = area under the receiver operating characteristic curve; SD = standard deviation. Positive-class F1 refers to the F1 score for the overweight/obesity class.
